# The differences of gonadal hormones and uterine transcriptome during shell calcification of hens laying hard or weak-shelled eggs

**DOI:** 10.1186/s12864-019-6017-2

**Published:** 2019-09-11

**Authors:** Jiacai Zhang, Yanan Wang, Cong Zhang, Mingxin Xiong, Shahid Ali Rajput, Yun Liu, Desheng Qi

**Affiliations:** College of Animal Nutrition and Feed Science, Huazhong Agricultural University, Wuhan, 430070 Hubei China

**Keywords:** Gonadal hormone, Transcriptome, Eggshell, Laying hen, Ion transport

## Abstract

**Background:**

Eggshell breaking strength is critical to reduce egg breaking rate and avoid economic loss. The process of eggshell calcification initiates with the egg entering the uterus and lasts about 18 h. It follows a temporal sequence corresponding to the initiation, growth and termination periods of shell calcification. During each period of shell calcification, our study investigated the differences of gonadal hormones and uterine transcriptome in laying hens producing a high or low breaking strength shell.

**Results:**

60 Hy-line Brown laying hens were selected and divided into two groups according to eggshell breaking strength. Eggshell breaking strength of 44.57 ± 0.91 N and 26.68 ± 0.38 N were considered to be the high strength group (HS) and low strength group (LS), respectively. The results showed that mammillary thickness and mammillary knob width of eggshells were significantly lower in the HS. Serum progesterone (P_4_) and 1,25-dihydroxy vitamin D_3_ [1,25-(OH)_2_D_3_] were significantly higher in the HS compared to the LS during the initiation period of calcification. Serum estradiol (E_2_) and calcium did not change significantly. All factors mentioned above had no significant differences in the growth and termination periods of calcification. The relative expression of CaBP-D_28k_ and PMCA 1b were not significantly different between HS and LS. The relative expression of NCX1 was significantly higher in HS compared to LS. Moreover, 1777 differentially expressed genes (DEGs) were obtained in the initiation period of calcification. However, few DEGs were identified in the growth or termination periods of calcification. 30 DEGs were selected as candidate genes involved in eggshell calcification during the initiation period of calcification by the analysis of GO terms and KEGG pathways.

**Conclusions:**

Our study concluded that mammillary thickness and mammillary knob width of the HS were significantly lower than LS. P_4_ and 1,25-(OH)_2_D_3_ were significantly higher in the initiation period of HS. They may impact initial calcification when the mammillary layer is formed. The initiation period of calcification determined eggshell strength rather than the growth or termination periods. We inferred P_4_ or 1,25-(OH)_2_D_3_ may effect the ultrastructure of the mammillary layer by regulating the expression of uterine genes.

**Electronic supplementary material:**

The online version of this article (10.1186/s12864-019-6017-2) contains supplementary material, which is available to authorized users.

## Introduction

The eggshells of laying hens are a composite bioceramic material containing 95% calcium carbonate as polymorphic calcite and 3.5% organic matrix proteins [1]. The eggshell provides mechanical protection for embryonic development and prevents microbial invasion. The calcium needed for embryonic development is produced mainly by the eggshell. The most common problem of eggshells in production is the defect rate, which increases with the age of the hens. Eggshell quality is one of the most important concerns in the poultry industry, greatly affecting profits. Eggs with low breaking strength are easily damaged during collection, storage, and transportation. On average, broken eggs account for 7.5% [2], which can lead to significant economic losses. The eggshell is formed in the uterus which is a segment of the oviduct of laying hens. The process of eggshell formation is divided into three crucial periods: initiation of crystal growth, linear crystal growth, and termination of mineralization [3]. The eggshell consists of a bilayered membrane, mammillary layer, palisade layer, vertical crystal layer, and cuticle layer [4]. Many researchers have proposed that differences in eggshell ultrastructure exist between high and low strength eggshells [5–7]. The ultrastructure of eggshell is related to matrix proteins that regulate morphology, growth kinetics, and crystallographic orientation of calcite crystals [8, 9], such as ovocleidin-116 [10], ovocleidin-17 [11], ovocalyxin-32 [12], and ovocalyxin-36 [13]. These proteins have been shown to be involved in the biomineralization of the eggshell [14].

To improve eggshell quality, many studies have investigated calcium and trace elements in the diet. However, increasing dietary calcium levels did not improve eggshell quality [15]. Manganese enhanced the synthesis of glycosaminoglycan in the eggshell membrane, which contributed to eggshell strength [16]. Zinc is a component of the enzyme carbonic anhydrase, which is essential for the formation of calcium carbonate [17]. Zinc supplementation increased eggshell thickness rather than eggshell strength [18]. The simultaneous supply of zinc and manganese in the diet increased eggshell strength from 33.5 to 36.7 N in the late phase of the laying cycle (70 weeks of age), while it had no effect in young laying hens (35 weeks of age) [19]. This conclusion was supported by Świątkiewicz et al. [20]. Moreover, Kim et al. reported that magnesium increased the eggshell strength from 3.1 to 3.4 kg/cm^2^ in 72-week-old laying hens [21]. Furthermore, copper has also been shown to affect eggshell quality [22]. Copper promoted desmosine formation in the shell membrane by activating amine oxidase [23]. Although the data from these studies were statistically significant, the author believed that the improvement in eggshell quality was minor. The improved eggshell strength from supplemental trace elements is much lower than the eggshell strength obtained in young hens.

It has been reported that exogenous estrogen improved eggshell quality and progesterone injected prolonged the period of eggshell formation [24]. Progesterone inhibited calcium ion transport and the concentration of calbindin mRNA in the eggshell gland [25]. Another study reported that the eggshell thickness of domestic fowls was increased following administration of mifepristone, an anti-progesterone compound [26]. Interestingly, a high incidence of hens producing hard-shelled uterine eggs was observed following progesterone injection [27]. Thus, we hypothesized that gonadal hormones may impact eggshell strength. However, the mechanisms are unclear. This study investigated the differences in gonadal hormone levels and the gene expression profile of the uterus during the three pivotal mineralization periods (initiation, growth, and termination) of eggs with a high or low breaking strength.

## Results

### Mechanical property parameters of the eggshell

Mechanical property parameters of the eggshell are provided in Table 1. A transverse views of eggshell ultrastructure are shown in Additional file 1. Breaking strength, eggshell thickness, and shell ratio of the HS were significantly higher compared to the LS (*P* < 0.05). However, the egg shape index was not significantly different (*P* > 0.05). Compared with the LS, mammillary thickness, mammillary knob width, and mammillary layer ratio were significantly lower in the HS (*P* < 0.05). Furthermore, the effective thickness and effective layer ratio were significantly higher in the HS (*P* < 0.05). The eggs in the uterus of each calcification period (initiation, growth, and termination) and the laid egg are shown in Additional file 2.

**Table 1:** Mechanical property parameters of eggshell in LS and HS^1^

Parameters	LS	HS
Breaking strength, N	26.68 ± 0.38	44.57 ± 0.91^*^
Eggshell thickness, mm	0.359 ± 0.003	0.391 ± 0.003^*^
Egg shape index	1.29 ± 0.006	1.28 ± 0.006
Shell ratio, %	9.43 ± 0.001	10.25 ± 0.001^*^
Mammillary thickness, μm	99.09 ± 3.57	60.00 ± 1.63^*^
Mammillary knob width, μm	83.23 ± 4.20	56.82 ± 2.56^*^
Effective thickness, μm	239.09 ± 8.14	288.64 ± 5.06^*^
Mammillary layer ratio, %	29.41 ± 1.15	17.21 ± 0.35^*^
Effective layer ratio, %	70.59 ± 1.15	82.79 ± 0.35^*^

^1^Results are reported as the mean ± SEM

^*^Means within a row significantly differ compared to the LS (*P* < 0.05)

### Concentration of hormones and calcium in the serum

The levels of P_4_, E_2_, 1,25-(OH)_2_D_3_, and Ca^2+^ are shown in Table 2. Compared with the LS, the concentration of P_4_ and 1,25-(OH)_2_D_3_ in the HS were significantly higher in the initiation period but did not change significantly in the growth or termination periods. However, the blood calcium level in the LS in the initiation period was significantly higher compared to the HS. The concentration of E_2_ did not change significantly in the calcification period.

**Table 2:** The concentrations of gonadal hormones and calcium in serum from LS and HS^1^

Parameters	Period	LS	HS
P_4_, pmol/L	Initiation	752.55 ± 118.09	1401.63 ± 155.53^*^
	Growth	656.33 ± 100.56	605.23 ± 80.16
	Termination	751.30 ± 134.07	616.66 ± 93.66
E_2_, pg/mL	Initiation	251.17 ± 39.43	296.61 ± 59.70
	Growth	243.41 ± 39.72	166.82 ± 24.02
	Termination	335.46 ± 58.20	221.92 ± 28.17
1,25-(OH)_2_D_3_, ng/mL	Initiation	22.90 ± 4.13	40.22 ± 6.89^*^
	Growth	21.59 ± 3.78	19.20 ± 3.22
	Termination	24.26 ± 5.22	19.79 ± 3.77
Ca, μg/mL	Initiation	335.58 ± 9.74	261.29 ± 15.42^*^
	Growth	201.67 ± 17.64	196.81 ± 9.67
	Termination	266.98 ± 17.37	239.03 ± 10.41

^1^Results are reported as the mean ± SEM

^*^Means within a row significantly differ compared to the LS (*P* < 0.05)

### Expression levels of genes in the duodenum

**Fig. 1:**
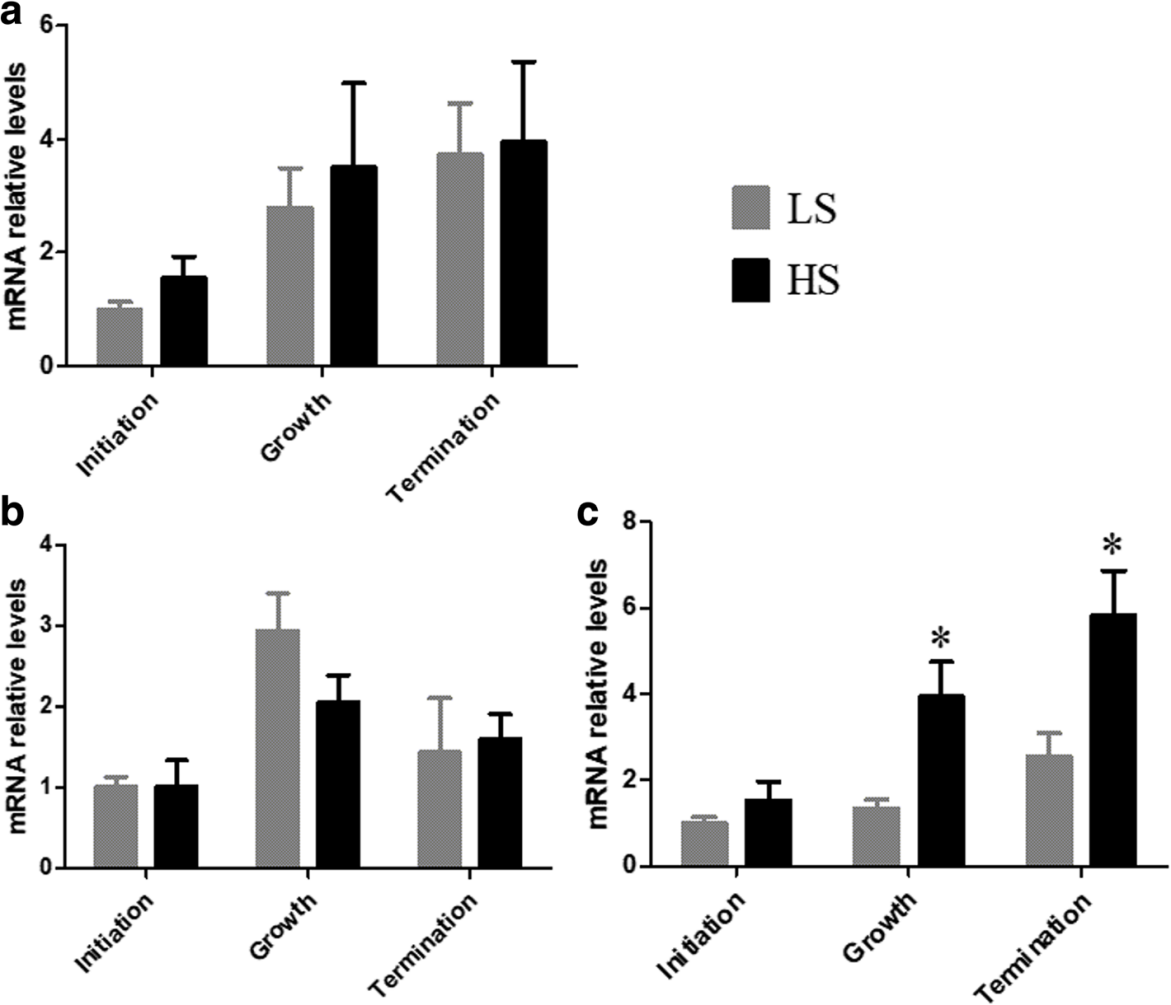
Relative expression levels of calcium ion transport-related genes in the duodenum. **a** PMCA1b; (**b**) CaBP-D_28k_; (**c**) NCX1. The initiation period of LS is set at 1.0. Values are means ± SEM. Means with * are obviously different from the LS in the same calcification period (*P* < 0.05)

Primers targeting CaBP-D_28k_, MPCA 1b, and NCX1 were designed by primer 5.0 (Additional file 3). The relative expression levels are shown in Fig. 1. These genes impact the translocation efficiency of calcium ions in the duodenum. No significant differences were apparent in the relative expression levels of PMCA 1b (Fig. 1a) and CaBP-D_28k_ (Fig. 1b) in the calcification period. In the growth and termination periods, the relative expression level of NCX1 (Fig. 1c) in the HS was significantly higher compared with LS. However, there was no difference in the expression of NCX1 in the initiation period.

### Differential transcriptome expression in the uterus

**Fig. 2:**
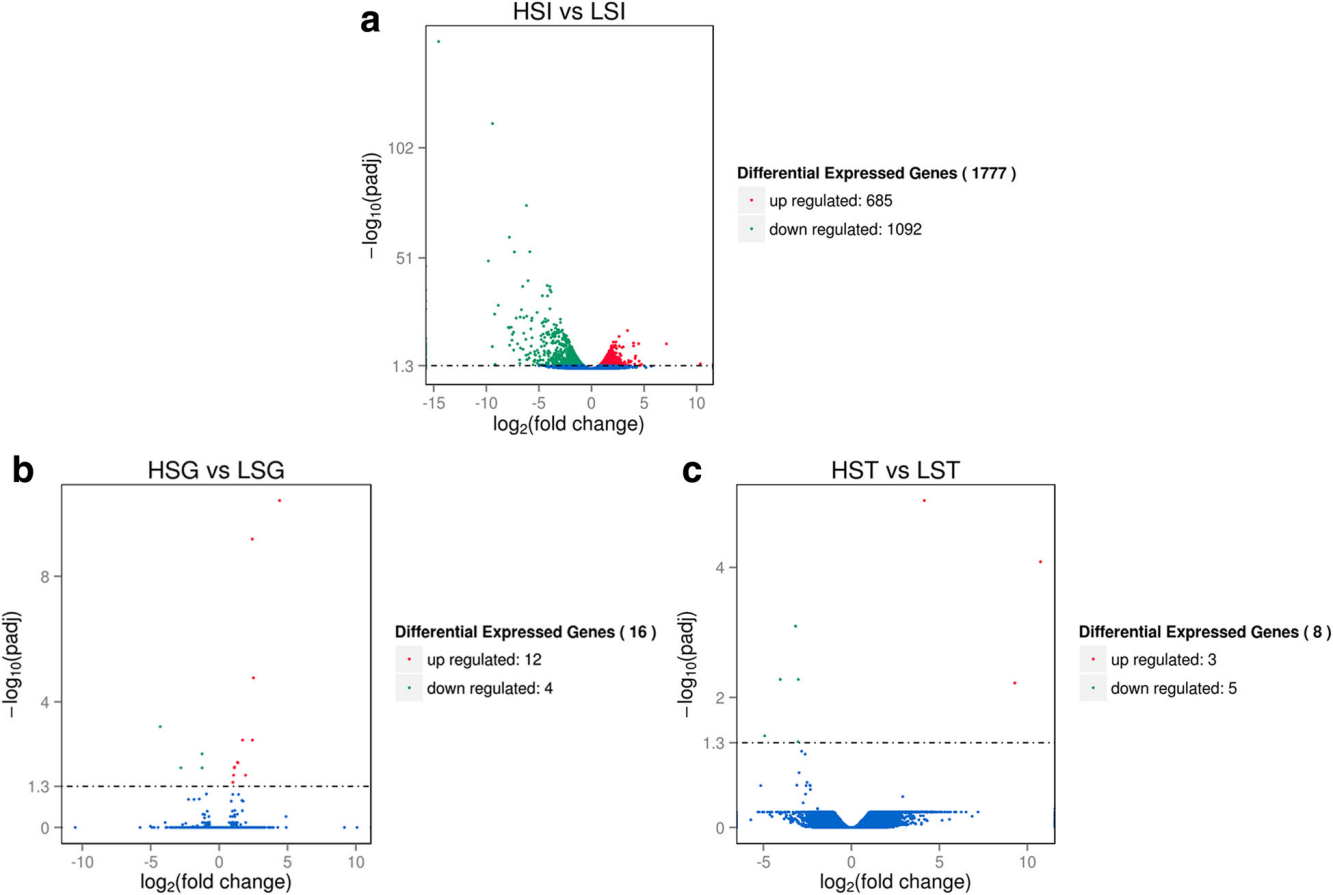
Volcano plot of differentially expressed genes (DEGs) in the uterus. **a** Initiation period of HS and LS (HSI vs. LSI); (**b**) Growth period of HS and LS (HSG vs. LSG); (**c**) Termination period of HS and LS (HST vs. LST)

The mRNA expression profile was investigated in the uterus of both HS and LS. DEGs were initially identified by fold change > 1.5 and *P* < 0.05 (Additional file 4). In the initiation period of calcification, 1777 annotated genes were significantly different in the uterus (Fig. 2a). Of the 1777 genes, 685 genes were upregulated and 1092 genes were downregulated (HSI vs. LSI). However, in the growth period of calcification, only 16 genes were identified as DEGs (Fig. 2b), which included 12 upregulated and 4 downregulated genes (HSG vs. LSG). A similar trend existed in the termination period of calcification in which eight genes were considered differentially expressed (Fig. 2c). Of these genes, three were upregulated and five were downregulated (HST vs. LST). From the results, it conclude that the initiation period of calcification is the most critical period for determining the mechanical properties of eggshells. Thus, in subsequent experiments, only the RNA-Seq data in the initiation period of calcification were analyzed.

**Fig. 3:**
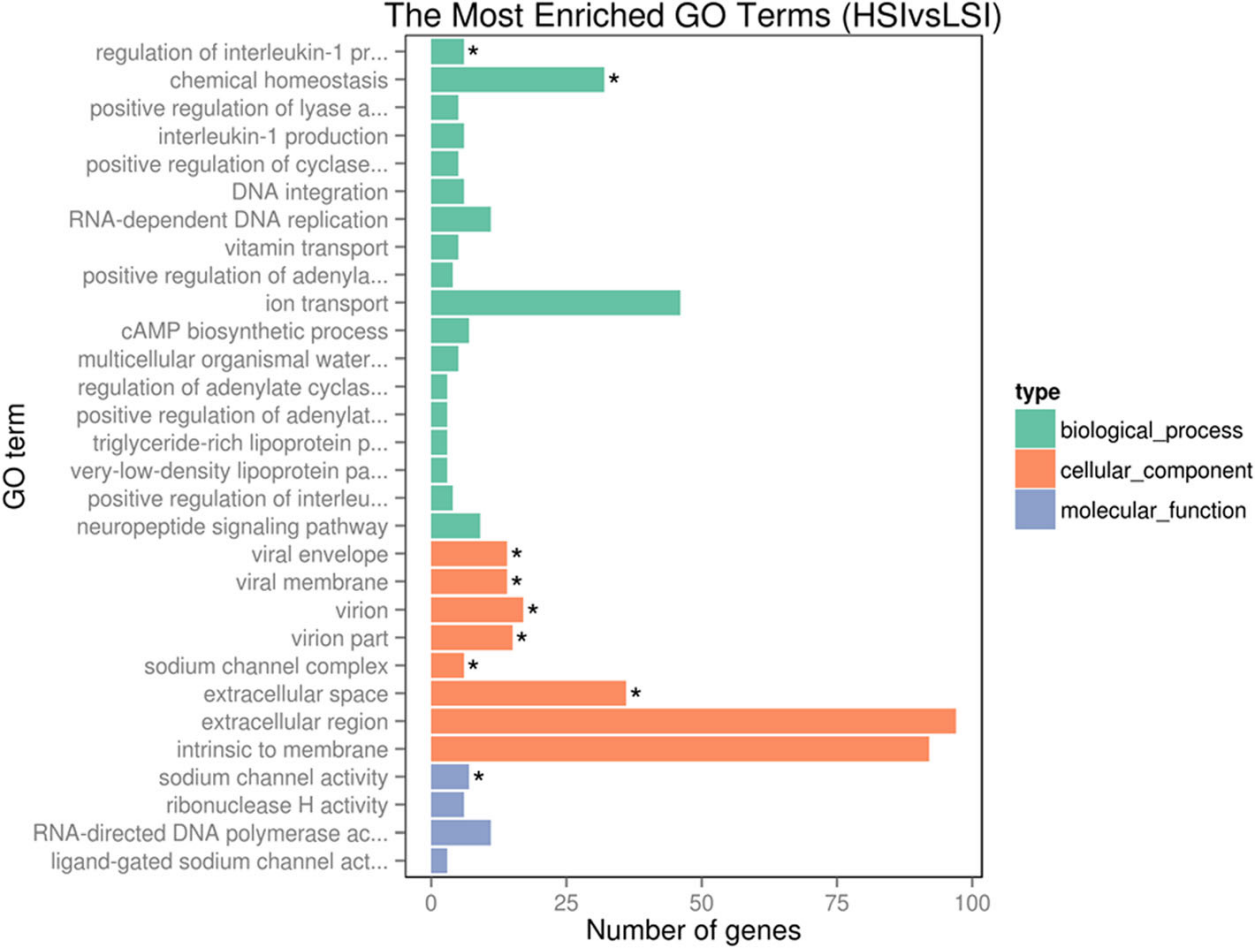
The enrichment of DEGs in GO terms during the initiation period of calcification (HSI vs. LSI). * means significantly different (*P* < 0.05)

DEGs of the initiation period were enriched in biological process, cellular component, and molecular function categories by GO analysis (http://www.geneontology.org/). GO terms with *P* < 0.05 were considered significantly enriched in DEGs. The GO term enrichment analysis showed that the highest enrichment of DEGs in biological process, cellular component, and molecular function were chemical homeostasis, extracellular space, and sodium channel activity, respectively (Fig. 3). DEGs were also plotted to KEGG reference pathways (https://www.kegg.jp/kegg/pathway.html); 87 KEGG pathways of chicken were assigned. The significantly enriched KEGG pathways (*P* < 0.05) are listed in Table 3. Of these KEGG pathways, the calcium signaling pathway has been reported to be linked to calcium metabolism in the uterus. In addition to calcium ions (Ca^2+^), bicarbonate ions (HCO_3_^−^) are also required for the calcification of eggshells. The expression of a large number of ion transporter genes was significantly different, while the pathway of ion transport from blood to uterine fluid of chicken was not identified in the KEGG pathway database. Therefore, we studied ion transport genes based on previous reports [28–30]. A total of 30 relevant DEGs were selected and some were used to confirm the accuracy of the RNA-Seq data. The functions of the selected genes are listed in Table 4.

**Table 3:** Significantly enriched KEGG pathways in the initiation period of calcification

Term	Gene number	Background number	*P*-value
Linoleic acid metabolism	6	28	0.0002
Glycerophospholipid metabolism	9	86	0.0009
Alpha-linolenic acid metabolism	5	25	0.0011
Drug metabolism, cytochrome P450	5	29	0.0019
Ether lipid metabolism	5	39	0.0059
Arachidonic acid metabolism	5	48	0.0130
Metabolism of xenobiotics by cytochrome P450	4	32	0.0148
Calcium signaling pathway	10	162	0.0166
GnRH signaling pathway	6	77	0.0230
Vascular smooth muscle contraction	7	100	0.0239
Glycosphingolipid biosynthesis, lacto and neolacto series	3	21	0.0250
Phagosome	8	127	0.0277
MAPK signaling pathway	11	214	0.0382

**Table 4:** Functions of DEGs related to calcification in the initiation period

Gene ID	Gene symbol	Log_2_(fold-change)	Gene description
ENSGALG00000000703	SLC45A3	−1.20	myelin-enriched sugar transporter [46]
ENSGALG00000010187	SLC1A1	− 1.27	glutamate transporter [47]
ENSGALG00000003582	SLC1A3	1.03	glutamate transporter [48]
ENSGALG00000005933	SLC5A11	1.13	sodium-dependent cotransporter [49]
ENSGALG00000006194	SLC52A3	−1.07	riboflavin transporter [50]
ENSGALG00000007998	SLC26A4	−7.57	chloride-iodide transport protein [51]
ENSGALG00000008844	SLC31A1	−1.34	copper transporter [52]
ENSGALG00000014372	SLC34A2	−4.21	phosphate transporter [53]
ENSGALG00000011003	SLC35F3	1.86	thiamine transporter [54]
ENSGALG00000017032	SLC25A15	1.94	mitochondrial ornithine carrier [55]
ENSGALG00000027743	SLC4A1	2.32	Bicarbonate transporter [56]
ENSGALG00000028756	SLC4A2	0.96	Bicarbonate transporter [57]
ENSGALG00000044387	SLC4A9	2.11	Bicarbonate transporter [58]
ENSGALG00000008045	SLC9A8	0.88	Sodium/proton exchangers [59]
ENSGALG00000001314	PTGS1	−1.02	Catalyse prostaglandin formation
ENSGALG00000006453	TF	−2.20	Regulate crystal size [45]
ENSGALG00000012869	OVAL	−3.65	Regulate crystal size [45]
ENSGALG00000023622	AVD	−2.81	Binding biotin
ENSGALG00000010926	SPP1	−1.64	Regulate crystal growth [60]
ENSGALG00000002207	ATP6V1G3	1.52	Proton pump
ENSGALG00000015868	ATP6V0D2	1.55	Proton pump
ENSGALG00000016446	ATP6V1C2	0.92	Proton pump
ENSGALG00000011258	ATP2B1	−0.86	Plasma membrane calcium transporter
ENSGALG00000007179	ATP13A5	2.23	Ca^2+^ homeostasis [61]
ENSGALG00000015454	CA8	1.56	Catalyse HCO_3_^−^ formation
ENSGALG00000006202	SCNN1B	1.51	Epithelial sodium channel [28]
ENSGALG00000006270	SCNN1G	1.25	Epithelial sodium channel [28]
ENSGALG00000014244	SCNN1A	2.07	Epithelial sodium channel [28]
ENSGALG00000009324	CFTR	1.22	Chloride channel [28]
ENSGALG00000025804	CLCN2	1.52	Chloride channel [28]

### RT-qPCR analysis

The relative expression of 12 genes, evaluated by RT-qPCR, was used to validate the RNA-Seq data. The primers used are listed in Additional file 5. Pearson correlation analysis was used and the Pearson coefficient was R = 0.920 with significant correlation (*P* < 0.0001), which was evaluated using log_2_ fold changes of RNA-Seq values (Additional file 6). The analysis confirmed the accuracy and reproducibility of the RNA-Seq results.

## Discussion

When eggshells are calcified in the uterus, large amounts of calcium ions and matrix proteins are required. Calcium metabolism and uterine proteins differ during the calcification periods (initiation, growth, and termination). Eggshell quality is determined by its ultrastructure [31], especially the palisade layer [32]. Therefore, many studies have focused on the growth period when the palisade layer is formed [29, 33]. However, the mammillary layer, which is formed during the initiation period of calcification, is also closely related to eggshell quality [34]. Therefore, our research was carried out in the initiation (HSI and LSI), growth (HSG and LSG), and termination (HST and LST) periods. Hormones related to calcium metabolism were detected in the blood. Differences in gene expression were identified in the uterus of the HS and LS. In the present study, compared with the LS, mammillary thickness and mammillary knob width in the HS were significantly lower, which is consistent with the previous report [6]. This indicated that the initiation period of calcification, as well as the growth period, is important for eggshell quality. The quality and ultrastructure of eggshells indicated the validity of grouping according to eggshell strength. The different appearance of eggs removed from the uterus after different oviposition times by manual dissection indicated the validity of the three calcification periods.

### Serum hormones related to calcium metabolism

1,25-(OH)_2_D_3_ is synthesized from 25-(OH)D_3_ in the kidney through the catalysis of 1α-hydroxylase and regulates duodenal calcium absorption. The laying hens metabolize sufficient 1,25-(OH)_2_D_3_ from dietary vitamin D_3_ to maintain shell quality [25]. Hens forming uncalcified shells synthesized less 1,25-(OH)_2_D_3_ [35]. In previous report, 1,25-(OH)_2_D_3_ increased in the growth period of calcification [36]. However, our results showed 1,25-(OH)_2_D_3_ increased in the HS during the initiation period of calcification. Moreover, serum levels of calcium in the initiation period of LS were significantly higher, which may be a result of inhibition in calcium ion transport.

P_4_ and E_2_ are key reproductive hormones for ovulation. Serum P_4_ showed the peak level at 4–6 h before ovulation and then quickly declined to basal level [37]. Similarly, serum E_2_ also showed the peak level at 2–6 h before ovulation and then returned to basal level [38]. A few studies have shown that P_4_ and E_2_ are related to eggshell quality. Eggshell thickness has been increased by E_2_ injection [25]. However, Bar et al. believed the effect of E_2_ on the synthesis of calbindin mRNA in the eggshell gland were minor. Oral administration of mifepristone (RU38486), an anti-progesterone compound, increased eggshell thickness [26]. On the other hand, the effect of P_4_ injection on eggshell calcification was related to oviposition time. P_4_ injected increased shell weight 4 or 10 h after oviposition and shell weight declined 16 h after oviposition [39]. Our results showed that P_4_ level of the HS were significantly higher than the LS, suggesting that P_4_ may regulate eggshell quality during the initiation period of calcification. However, E_2_ did not differ significantly during any calcification periods.

### Ca^2+^ transport in the duodenum

Dietary calcium is primarily absorbed in the duodenum. The calcium level has a great influence on eggshell quality. Eggshell quality increased when dietary calcium was increased from 1.5 to 2.5%, while no significant differences were observed in these variables between 2.5 and 3.5% calcium levels [40]. This is consistent with the results of Swiatkiewiz et al., in which eggshell quality parameters were not improved significantly when dietary calcium was increased from 3.2 to 4.2% [15]. These studies demonstrated that eggshell quality was improved by increasing the calcium level in calcium deficient diets rather than in adequate calcium diets. Our study examined the relative expression levels of genes in the duodenum involved in calcium ion absorption, including calbindin (CaBP-D_28k_), Na^+^/Ca^2+^ exchanger (NCX1), and ATPase plasma membrane Ca^2+^ transporter (PMCA1b). CaBP-D_28k_ and PMCA1b were not differentially expressed in the same calcification period. This indicated that the absorption of Ca^2+^ in the duodenum had no significant effect on eggshell quality. This was similar to the conclusion of Yosef et al. whose research showed that duodenal CaBP-D_28k_ expression of aged hens (675 and 645 days, respectively) was not significantly different from that of young hens (307 and 245 days, respectively) [41]. Furthermore, eggshell quality was improved after moulting, while duodenal CaBP-D_28k_ expression did not exhibit a significant change. Although some studies have proposed different views [42], more researchers support the notion that duodenal CaBP-D_28k_ expression does not effect eggshell quality [43]. In the growth and termination periods of calcification, NCX1 was significantly higher in the HS while had no significant change in the initiation period. This is likely a result of the large amount of Ca^2+^ needed in the growth and termination periods of calcification.

### Uterine gene expression in the initiation period

Eggshells show a highly ordered structure from deposition of CaCO_3_ and matrix proteins. A large amount of Ca^2+^ and HCO_3_^−^ are required to form CaCO_3_. Matrix proteins regulate the calcite crystal structure and are secreted by uterine glandular cells into the uterine fluid. Ca^2+^ and HCO_3_^−^ are transported from the blood to the uterine fluid through uterine glandular cells by ion transporter proteins. The transcriptome of uterine tissue during the calcification period has been described in previous studies [28, 29]. This transcriptome compared gene expression in the uterus collected during the active calcification phase (growth period of calcification), when there is rapid secretion and growth of CaCO_3_ leading to the formation of the compact shell layer versus the uterus with the absence of egg. However, differences in the uterine transcriptome from hens laying eggs with high and low eggshell breaking strength during synchronous calcification periods have not been reported. The current study identified DEGs in the uterus obtained according to eggshell breaking strength. Few DEGs were observed during the growth and termination periods of calcification, while 1777 genes were differentially expressed during the initiation period. These results suggest that the initiation period have a greater contribution to eggshell strength than the other two periods of calcification. Therefore, we focused our attention on the initiation period and screened 30 DEGs as candidates related to calcification according to KEGG analysis and previous studies (Table 4).

The selected candidate genes were broadly classified into two types: extracellular proteins secreted into uterine fluid (TF, OVAL, etc.), and ion transporter genes in glandular cells (SLC31A1, SLC31A2, etc.). Marie et al. identified 308 proteins in uterine fluid during the initiation, growth, and termination periods of calcification and obtained nine proteins related to calcification in the initiation period, including TF and OVAL [3]. TF and OVAL were also reported to be related to eggshell structure in previous study [44]. This is consistent with the study which TF and OVAL were associated with the structural organization of the mammillary layer and crystal size by genetic associations [45]. In the present study, the gene expression levels of TF and OVAL were significantly higher in the LS. This implied that they adversely affect the formation of the mammillary layer and reduce eggshell strength. We also observed that the osteopontin (SPP1) gene was significantly upregulated in the LS as compared to HS, which may imply SPP1 damaged eggshell quality. A previous study demonstrating that SPP1 inhibit the growth of calcite crystals in vitro supports this view [60].

**Fig. 4:**
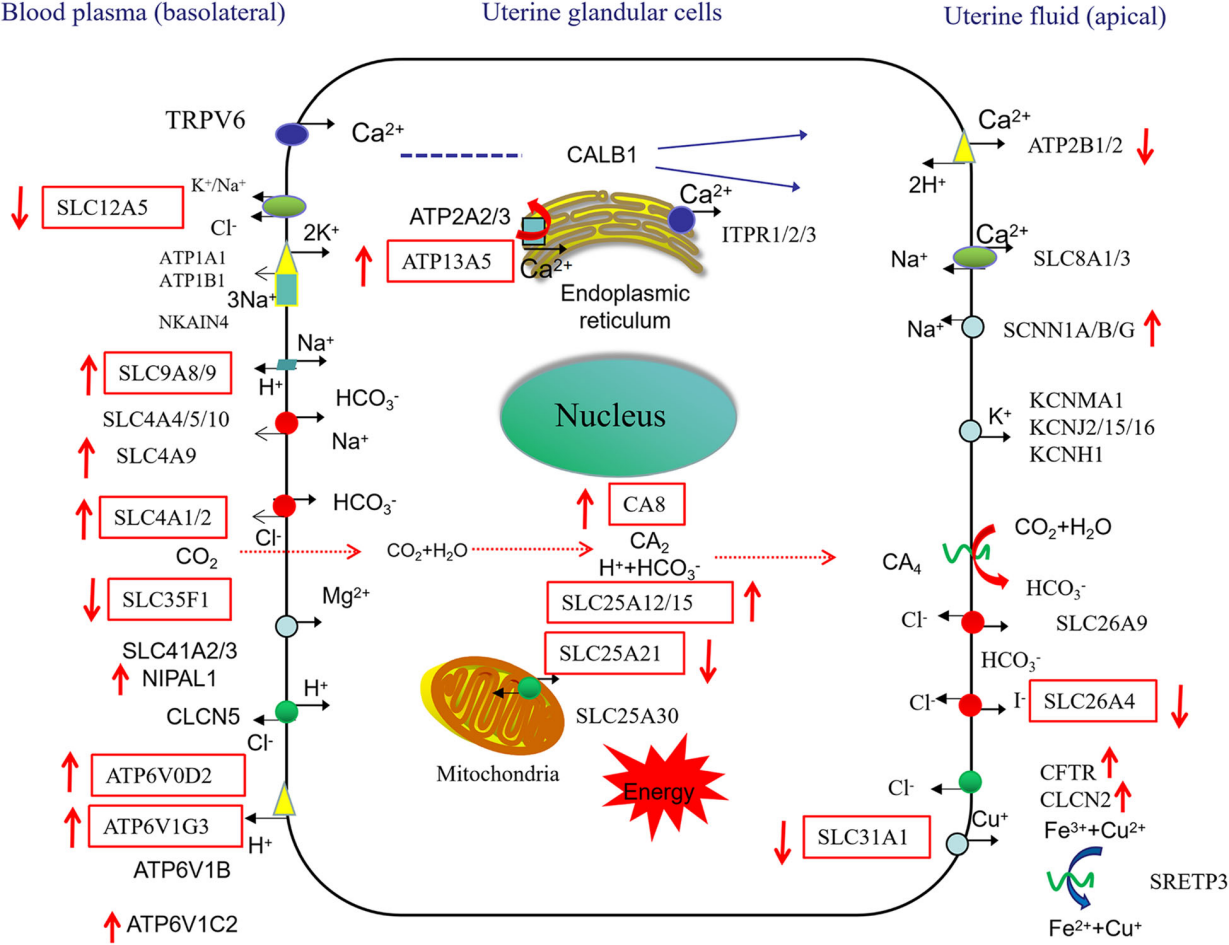
The updated model of ion transporters in the uterus during the initiation calcification of eggshell. Red squares indicate novel DEGs identified in our study; Upward arrows indicate upregulated genes and downward arrows indicate downregulated genes (HSI vs. LSI)002E

GO analysis revealed that many DEGs were enriched in ion transport functions associated with eggshell calcification (Fig. 3). When the eggshell is calcified, it is necessary to maintain sufficient ions in the uterus. Jonchère et al. identified uterine ion transporters in uterine tissue where calcification occurred and when calcification was prevented artificially by expelling the egg during 4 consecutive days previously [28]. A total of 37 ion transport genes were selected and a model of ion transport in uterine glandular cells was provided. The model of ion transport was updated and enriched in later research using the same method [29]. The above studies did not involve eggshell strength, and Ca^2+^ and 1,25(OH)_2_D_3_ levels in the blood were affected by expelling the egg. We identified differences in ion transport of the uterus producing high and low breaking strength eggshells during normal calcification. Our aim was to identify the genes affecting eggshell strength. In addition to the common ion transport genes identified in the previous reports, the present study has also described some novel genes involved. The uterine ion transport model was further updated based on the previous studies (Fig. 4).

Ca^2+^ in uterine fluid is derived from calcium in the blood. TRPV6, CALB1, and ATP2A2/3, which are responsible for Ca^2+^ transport, were not significantly different between groups. However, the endoplasmic reticulum calcium transporter (ATP13A5) was significantly upregulated in the HS as compared to LS. HCO_3_^−^ in uterine fluid is generated by CAs catalyzing reversible CO_2_ hydration, and transported to uterine fluid. Immunohistochemical studies have indicated that CAs are present in chicken uterine glandular cells [62] and cytosolic CA 2–14 of *Gallus gallus* has been referenced in the gene database (NCBI). We observed a high expression level of the CA8 gene in the HS, but no differences in other CAs, suggesting that CA8 may play a pivotal role in the conversion of intracellular CO_2_ to HCO_3_^−^ in uterine glandular cells during the initiation period of calcification. Our study showed that the expression levels of the HCO_3_^−^ transporters SLC4A1, SLC4A2, and SLC4A9 were significantly upregulated in the HS as compared to the LS. This indicates that the supply of HCO_3_^−^, rather than Ca^2+^, affected eggshell quality, even in the LS. This may also explain why eggshell quality could be improved by adding NaHCO_3_ to the diet [63], rather than increasing dietary calcium levels [15]. During eggshell formation, progressive acidification of uterine fluid and uterine glandular cells occurs. The production of HCO_3_^−^ is accompanied by the appearance of H^+^, and the pH in the uterine fluid is lowered by the generation of H^+^ [64], which inhibits CaCO_3_ deposition. H^+^ is transported by the proton pumps (ATP6V0D2, ATP6V1G3, ATP6V1B, and ATP6V1C2) to maintain intracellular pH [65]. The relative expression levels of the proton pumps were significantly upregulated in the HS as compared to the LS, which indicated the ability of the HS to exclude H^+^ was higher than that of the LS.

## Conclusions

In conclusion, our study supported the previous view that mammillary thickness, mammillary knob width, and mammillary layer ratio are significantly lower in the HS. Serum P_4_ and 1,25-(OH)_2_D_3_ levels were higher during the initiation period of calcification of the HS. Serum E_2_ and calcium had minor effect on eggshell strength. The large differences in the transcriptome proved that the initiation period of calcification determines eggshell strength. The uterine ion transport model was further updated based on previous studies, improving our understanding of the eggshell calcification process.

## Materials and methods

### Ethical statement and experimental procedures

The experimental animal procedure was approved by the Scientific Ethics Committee of Huazhong Agricultural University on 11 May 2017. The ethical approval code is HZAUCH-2017-006. A total of 210 42-week-old Hy-Line Brown laying hens were caged individually and subjected to a 16 h:8 h light:dark cycle. Hens were fed a layer mash ad libitum as recommended by NRC (1994). The oviposition time per hen was observed and recorded from 5:00 am to 5:00 pm for 10 consecutive days named observation period, which contributed to determine the calcification periods. During the observation period, the eggshell breaking strength of eggs from each hen was determined every day, which contributed to select the hens laid eggs with hard or weak-shell. The laying hens with minor fluctuations in oviposition time (the fluctuation less than 30 min) and eggshell breaking strength (the fluctuation less than 4 N) were remained and others were discarded. In the remaining laying hens, 30 laying hens laid hard-shelled eggs were selected as high breaking strength group (HS) and 30 laying hens laid weak-shelled eggs were selected as low breaking strength group (LS). The eggshell breaking strength in HS and LS were 44.57 ± 0.91 N and 26.68 ± 0.38 N (mean ± SD), respectively. The entire process of eggshell calcification in uterus was divided into three calcification periods of 6–8 h (initiation), 14–16 h (growth), and 20–22 h (termination) after oviposition according to the previous study [3]. 10 hens in each group selected randomly were sampled in each calcification period.

### Sample collection

Three eggs per hen were collected. Blood samples were taken from the wing vein for the three calcification periods of each group. The hens were slaughtered after blood samples were taken. The duodenum, kidney, and uterus were surgically removed, frozen in liquid nitrogen immediately, and stored at − 80 °C. The blood samples were centrifuged (Eppendorf centrifuge 5804R, Hamburg, Germany) at 1000×*g* at 4 °C for 10 min, and the serum was separated and stored at − 80 °C for analyzing levels of calcium, estrogen (E_2_), progesterone (P_4_), and 1,25-dihydroxy vitamin D_3_ [1,25-(OH)_2_D_3_] levels.

### Mechanical properties and ultrastructure of eggshells

Eggshell breaking strength, eggshell thickness, egg weight, and egg shape index were assessed. The length, width, and eggshell thickness of eggs were determined using an electronic digital micrometer (Shanghai Shenhan Measuring Tools Co., Ltd., Shanghai, China). Eggshell breaking strength was determined using an Eggshell Force Gauge (EFG-0503, Robotmation Co., Ltd., Japan). Eggs and eggshells were weighed using an electronic balance (AUY220, Shimadzu Corporation, Kyoto, Japan). The egg shape index was calculated as length/width and the shell ratio was calculated as eggshell weight/egg weight × 100%. A total of 10 eggs from each group were selected randomly for scanning ultrastructures using a scanning electron microscope (JSM-6390LV, JEOL Ltd., Tokyo, Japan). The selected eggs were broken manually after being washed with distilled water to remove dirt on the outer surface. The contents of the interior were discarded and the inside of the shell was cleaned with distilled water to remove residual egg white. A piece (1 cm^2^) of each eggshell was cut from the equatorial region. The eggshell membranes were removed as the previous method reported by Kaplan et al. [66] and used by Gongruttananun et al. [67]. The eggshell fragments without membranes were coated with gold powder and the transverse surface was imaged.

### Detection of hormones and calcium levels in the serum

The concentrations of E_2_, P_4_, and 1,25-(OH)_2_D_3_ in the serum were measured using enzyme-linked immunosorbent assay kits (Nanjing Jiancheng Bioengineering Institute, Nanjing, China). The measuring procedures were performed according to the manufacturer’s instructions. Serum calcium was detected by atomic absorption spectrophotometer (AA-6300C, Shimadzu Corporation).

### RT-qPCR of genes related to calcium translocation in the duodenum

The relative expressions of calbindin (CaBP-D_28k_), Na^+^/Ca^2+^ exchanger (NCX1), and ATPase plasma membrane Ca^2+^ transporter (PMCA1b) were used to verify the capacity of calcium absorption in the duodenum. These genes are responsible for calcium ion transport. The primers for RT-qPCR were designed by primer 5.0. Total mRNA was extracted from the uterus with TRIzol reagent (Invitrogen, Carlsbad, CA, USA) according to the manufacturer’s instructions. mRNA quality and concentration were determined using a nucleic acid concentration analyzer (NanoDrop 2000, Thermo Fisher Scientific, Waltham, MA, USA) at 260 and 280 nm. Reverse transcription to synthesize the cDNA library was performed using the PrimeScriptTM RT reagent kit (TaKaRa, Japan). Expression levels of candidate genes were analyzed using RT-qPCR analyzer (CFX384, Bio-Rad, Hercules, CA, USA) with SYBR Green Dye (Bio-Rad, USA). After the initial temperature rise to 95 °C, denaturation was performed at 95 °C for 5 min; all reactions were then subjected to 40 cycles at 95 °C for 5 s, annealing at the appropriate annealing temperature for 30 s, and extension at 72 °C for 20 s. CT values were normalized using the reference gene (β-actin). The fold change was calculated using the 2^-ΔΔCT^ method [68].

### Transcriptomics analysis of the uterus

The uterus samples of the HS and LS were prepared for high throughput sequencing. There were three uterus samples from each calcification period per group, namely, the initiation period in the HS or LS (HSI and LSI, respectively), the growth period in the HS or LS (HSG and LSG, respectively), and the termination period in the HS or LS (HST and LST, respectively). Total mRNA was isolated with TRIzol reagent (Invitrogen). The concentration, quality, and integrity of mRNA were determined using a NanoDrop spectrophotometer (NanoDrop Technologies, Wilmington, DE, USA). RNA library construction and sequencing were performed at Shanghai Personal Biotechnology Co., Ltd. (Shanghai, China). The cDNA libraries were constructed following the TruSeq RNA Sample Preparation Guide (Illumina, San Diego, CA, USA). Poly (A) mRNA was isolated from purified total RNA using biotin-oligo (dT) magnetic beads and fragmented to generate average insert sizes of approximately 350 bp before creating the cDNA libraries. Quality control was conducted using PicoGreen fluorescence spectrophotometry and an Agilent Bioanalyzer (Agilent Technologies, Palo Alto, CA, USA). A cluster was generated, diluted to 4–5 pM, and sequenced using the Illumina NextSeq 500 System with paired-end 2 × 150-bp reads.

### Validation of differentially expressed genes (DEGs)

To confirm the accuracy of the RNA sequencing (RNA-Seq) gene expression data obtained from high throughput sequencing, RT-qPCR was carried out on the 12 selected genes that were considered as candidate genes related to eggshell calcification. RT-qPCR was performed as described above.

### Statistical analysis

All values were analyzed by one-way ANOVA analysis of variance followed by Duncan test. Values were expressed as mean ± standard error of the mean (SEM) and the analyses were conducted using IBM SPSS Statistics 20 (IBM Corporation, Armonk, NY, USA). *P* values < 0.05 were considered statistically significant. The figures were generated using GraphPad Prism 5 (Graph Pad Software Inc., San Diego, CA, USA). Raw RNA-Seq reads were preprocessed, assembled and filtered according to the description in the previous study [69]. Reference genome and gene model annotation files were downloaded from genome website (http://asia.ensembl.org/index.html). Index of the reference genome was built using Bowtie (v2.0.6) [70] and paired-end clean reads were aligned to the reference genome using TopHat (v2.0.9) [71]. HTSeq (v0.6.1) was used to count the reads numbers mapped to each gene [72] and then the reads per kilo base per million reads (RPKM) of each gene was calculated based on the length of the gene and reads count mapped to this gene [73]. Differential expression analysis was performed using the DESeq software (1.10.1) [74]. The resulting *P*-values were adjusted using the Benjamini and Hochberg’s method for controlling the false discovery rate [75]. Genes with fold change > 1.5 and *P*-value < 0.05 were identified as DEGs. Gene Ontology (GO) enrichment analysis of DEGs was implemented by the GOseq software (1.10.0) [76], in which gene length bias was corrected. GO terms with corrected *P*-value less than 0.05 were considered significantly enriched by DEGs. KEGG database (http://www.genome.jp/kegg/) and KOBAS software (v2.0.12) were used to test the statistical enrichment of DEGs in KEGG pathways [77].

## Additional files

Additional file 1: Scanning electron microscope images showing the transverse view of eggshellultrastructure from different breaking strength. Figure showing effective layer, mammillary layer, mammillary knob in HS and LS. (PDF 454 kb)

Additional file 2: The eggs in different calcification periods. Figure showing the differences of eggshell during initiation, growth and termination periods respectively. (PDF 510 kb)

Additional file 3: The primer sequences of target genes for RT-qPCR in duodenum. Word file giving the primer sequences. (DOC 32 kb)

Additional file 4: DEGs in hen uterus during different period of calcification. Excel file describing the DEGs that were annotated and non-annotated. (XLS 580 kb)

Additional file 5: Primer sequences of candidate genes for RT-qPCR. Word file giving the primer sequences. (DOC 50 kb)

Additional file 6: Correlation of Log2(fold change) between RNAseq results (abscissa) and RT-qPCR results (ordinate). Figure showing the accuracy and reproducibility of the RNA-Seq results. (PDF 206 kb)

## Data Availability

The RNA-Seq datasets have been submitted to NCBI Sequence Read Archive (SRA). SRA accession: PRJNA523965.

## Abbreviations

1,25-(OH)_2_D_3_: 1,25-dihydroxy vitamin D_3_
DEGs: Differentially expressed genes
E_2_: Estrogen
GO: Gene Ontology
HS: High breaking strength group
HSG: Growth period of calcification in HS
HSI: Initiation period of calcification in HS
HST: Termination period of calcification in HS
KEGG: Kyoto Encyclopedia of Genes and Genomes
LS: Low breaking strength group
LSG: Growth period of calcification in LS
LSI: Initiation period of calcification in LS
LST: Termination period of calcification in LS
NRC: National research council
P_4_: Progesterone
RT-qPCR: Real time quantitative PCR
