# Supplementary material for: The differences of gonadal hormones and uterine transcriptome during shell calcification of hens laying hard or weak-shelled eggs
Source: BMC Genomics. 2019 Sep 11;20:707. doi: 10.1186/s12864-019-6017-2 (PMC6737649; doi:10.1186/s12864-019-6017-2)
Supplement: Supplementary file 1 — Scanning electron microscope images showing the transverse view of eggshellultrastructure from different breaking strength. Figure showing effective layer, mammillary layer, mammillary knob in HS and LS. (PDF 454 kb) [file 12864_2019_6017_MOESM1_ESM.pdf]

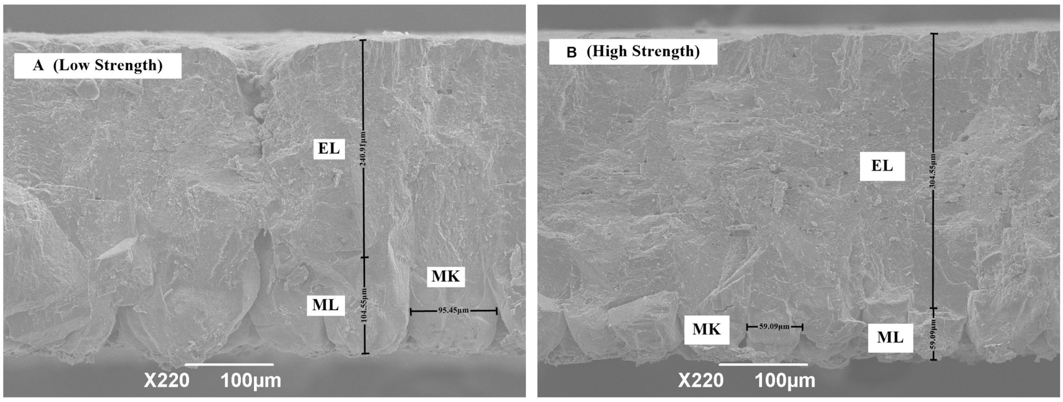

Supplemental Figure 1. Scanning electron microscope images showing the transverse view of eggshell ultrastructure from different breaking strength. (A) Eggshell of low breaking strength. (B) Eggshell of high breaking strength. EL: effective layer; ML: mammillary layer; MK:mammillary knob. Scale bar: 100µm.
