# Supplementary material for: The differences of gonadal hormones and uterine transcriptome during shell calcification of hens laying hard or weak-shelled eggs
Source: BMC Genomics. 2019 Sep 11;20:707. doi: 10.1186/s12864-019-6017-2 (PMC6737649; doi:10.1186/s12864-019-6017-2)
Supplement: Supplementary file 2 — The eggs in different calcification periods. Figure showing the differences of eggshell during initiation, growth and termination periods respectively. (PDF 510 kb) [file 12864_2019_6017_MOESM2_ESM.pdf]

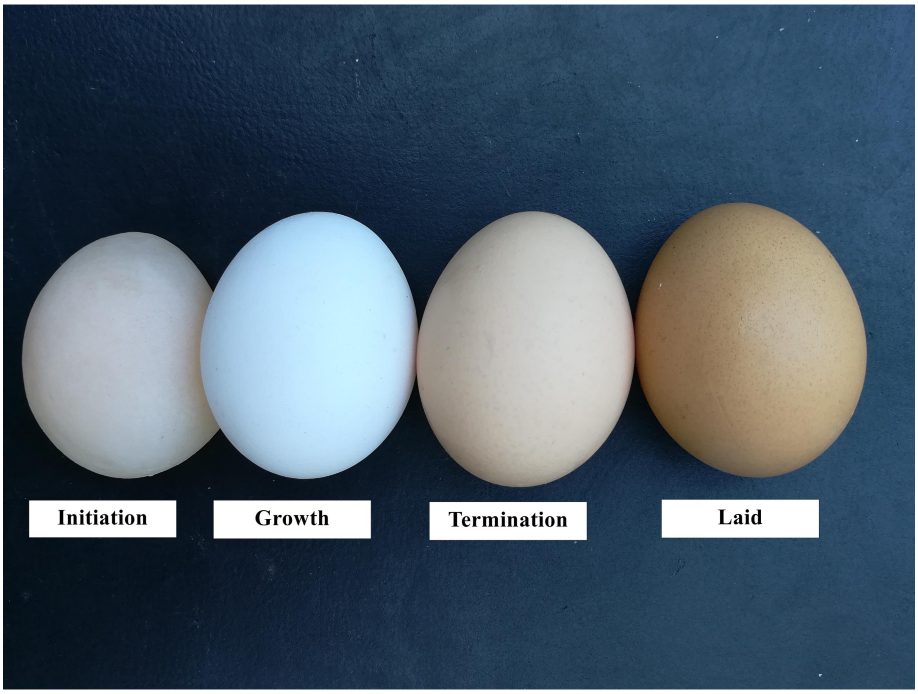

Supplemental Figure 2. The eggs in different calcification periods. Initiation: a thin layer of mineral cover the outer surface of the eggshell membrane; Growth: Hard eggshell with white color was formed; Termination: The appearance of eggshell showed light brown color; Laid: the egg wtih dark brown color
