# Supplementary material for: The differences of gonadal hormones and uterine transcriptome during shell calcification of hens laying hard or weak-shelled eggs
Source: BMC Genomics. 2019 Sep 11;20:707. doi: 10.1186/s12864-019-6017-2 (PMC6737649; doi:10.1186/s12864-019-6017-2)
Supplement: Supplementary file 3 — The primer sequences of target genes for RT-qPCR in duodenum. Word file giving the primer sequences. (DOC 32 kb) [file 12864_2019_6017_MOESM3_ESM.doc]

Supplemental Table 1. The primer sequences of target genes for RT-qPCR in duodenum

| Gene | Accession | Forward/Reverse Primer Sequences(5’-3’) | Fragment size (bp) | Annealing temperature (℃) |
| --- | --- | --- | --- | --- |
| CaBP-D_28k_ | EU404189 | F:TTAAATCTGCGTTGCTTCCATACA | 297 | 60 |
|  |  | R:GGCCCATCCTGCACTCCATAAC |  |  |
| PMCA 1b | XM_416133 | F: TTCAGGTACTCATGTGATGGAAGG | 98 | 60 |
|  |  | R:CAGCCCCAAGCAAGGTAAAG |  |  |
| NCX1 | DQ987923 | F: ACGGGAAATAACCATCAA | 323 | 60 |
|  |  | R: GCCAAGTGTAGGCAAAGA |  |  |
| β-action | L08165 | F:CATGCCATCCTCCGTCTG | 443 | 60 |
|  |  | R: AGGGCTGTGATCTCCTTCTG |  |  |
