# Supplementary material for: The differences of gonadal hormones and uterine transcriptome during shell calcification of hens laying hard or weak-shelled eggs
Source: BMC Genomics. 2019 Sep 11;20:707. doi: 10.1186/s12864-019-6017-2 (PMC6737649; doi:10.1186/s12864-019-6017-2)
Supplement: Supplementary file 5 — Primer sequences of candidate genes for RT-qPCR. Word file giving the primer sequences. (DOC 50 kb) [file 12864_2019_6017_MOESM5_ESM.doc]

Supplemental Table 2. Primer sequences of candidate genes for RT-qPCR

| Gene | Accession | Forward/Reverse Primer Sequences | Fragment size (bp) | Annealing temperature (℃) |
| --- | --- | --- | --- | --- |
| β-action | L08165.1 | F:CATGCCATCCTCCGTCTG | 443 | 58 |
|  |  | R:AGGGCTGTGATCTCCTTCTG |  |  |
|  |  |  |  |  |
| ATP2B1 | NM_001168002.3 | F:ATGTGCCTTGCTGGGTTT | 284 | 58 |
|  |  | R:CCATTGGCTCAATTACAGTTTT |  |  |
|  |  |  |  |  |
| SPP1 | U01844.1 | F:CAGCCCGCAGTAGGAGTT | 352 | 55 |
|  |  | R:CCAAGCAGGAGTCACAGA |  |  |
|  |  |  |  |  |
| SLC4A9 | XM_025154989.1 | F:CTGCGGAGCCTGAGGTAT | 296 | 55 |
|  |  | R:AATGAAGACGGGAAATGG |  |  |
|  |  |  |  |  |
| TF | NM_205304.1 | F:GCCCATTGCTGCTGAGAT | 213 | 55 |
|  |  | R:CCCGATTCTATGCCTTCC |  |  |
|  |  |  |  |  |
| PTGS1 | XM_425326.6 | F:GGAAGGCTATGAGTGCGACTG | 302 | 58 |
|  |  | R:GGAGGATGCGGGTGTAAT |  |  |
|  |  |  |  |  |
| SLC4A1 | NM_001290554.1 | F:GTGCCCATCTCCATCTTCGT | 266 | 58 |
|  |  | R:TTCACCAGCTTCCGCTCC |  |  |
|  |  |  |  |  |
| SLC4A2 | NM_204963.1 | F:CGTCCAACGGGCTGGAGTA | 365 | 58 |
|  |  | R:GAAGAAGGCGATGAAGAAGGTG |  |  |
|  |  |  |  |  |
| OVAL | AY223553.1 | F:GAGGCTTGGAACCTATCA | 154 | 55 |
|  |  | R:ATGGCATTAACCAGAACC |  |  |
|  |  |  |  |  |
| ATP13A5 | XM_422713.5 | F:CCAGAAATCCAGCCTTGT | 376 | 55 |
|  |  | R:TTCGGTCAGACTCATTGTTA |  |  |
|  |  |  |  |  |
| AVD | NM_205320.1 | F:TGCTCGCTGACTGGGAAAT | 196 | 58 |
|  |  | R:TGACGGTGAAGCCAAAGG |  |  |
|  |  |  |  |  |
| CLCN2 | XM_423073.4 | F:GGGGAATCTATGAGAACGAGG | 359 | 58 |
|  |  | R:CACAATCTTGCGGTTGAGGT |  |  |
|  |  |  |  |  |
| SCNN1A | NM_205145.2 | F:CGGTGGACAAGAACGACT | 200 | 55 |
|  |  | R:GCGGCAAGCATAAATAAA |  |  |
