# Supplementary material for: The differences of gonadal hormones and uterine transcriptome during shell calcification of hens laying hard or weak-shelled eggs
Source: BMC Genomics. 2019 Sep 11;20:707. doi: 10.1186/s12864-019-6017-2 (PMC6737649; doi:10.1186/s12864-019-6017-2)
Supplement: Supplementary file 6 — Correlation of Log2(fold change) between RNAseq results (abscissa) and RT-qPCR results (ordinate). Figure showing the accuracy and reproducibility of the RNA-Seq results. (PDF 206 kb) [file 12864_2019_6017_MOESM6_ESM.pdf]

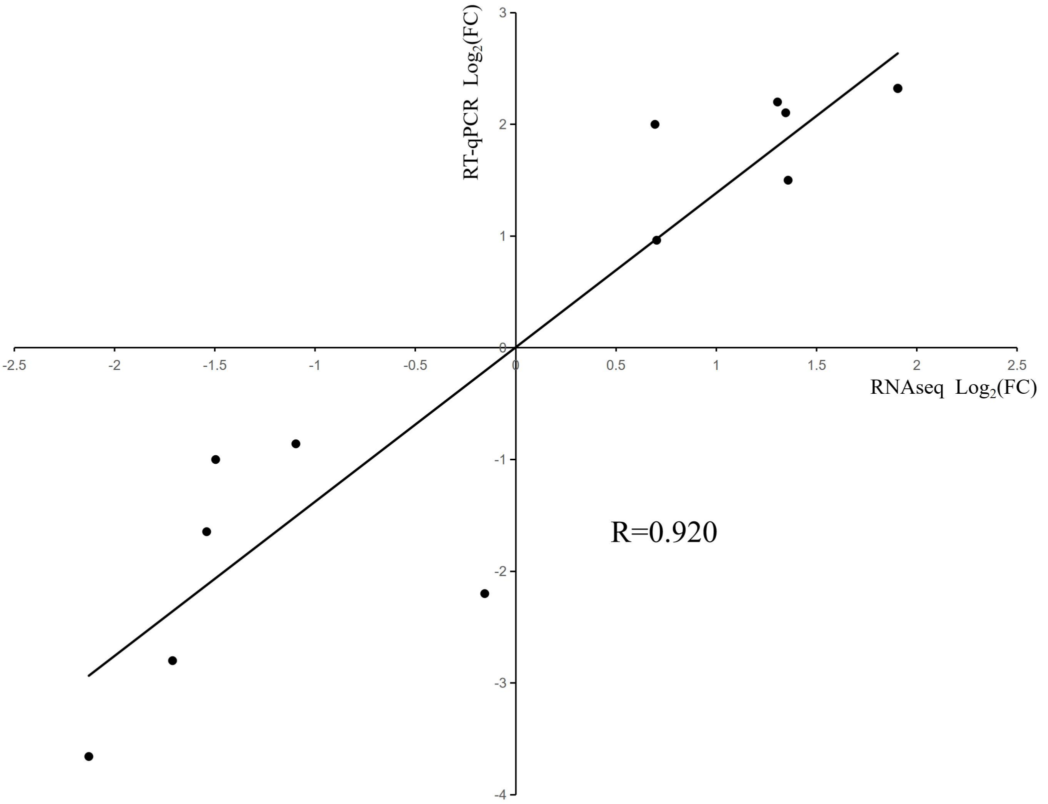

Supplemental Figure 3. Correlation of Log_2(fold change) between RNAseq results (abscissa) and RT-qPCR results (ordinate). Fold change of RT-qPCR correlated significantly (*P*<0.0001) with which of RNAseq for 12 genes. R=0.920.
